# 2140. INO-4800, a DNA Plasmid Vaccine Encoding the Ancestral SARS-CoV-2 Spike Protein, Is Stable Over a Range of Temperatures Not Requiring Ultra-Cold Chain Storage

**DOI:** 10.1093/ofid/ofac492.1760

**Published:** 2022-12-15

**Authors:** Robert J Juba, Anthony Samawova, Katherine Seals, Peggy Kinney, E J Brandreth

**Affiliations:** Inovio Pharmaceuticals, Plymouth Meeting, Pennsylvania; Inovio Pharmaceuticals, Plymouth Meeting, Pennsylvania; Inovio Pharmaceuticals, Plymouth Meeting, Pennsylvania; Inovio Pharmaceuticals, Plymouth Meeting, Pennsylvania; Inovio Pharmaceuticals, Plymouth Meeting, Pennsylvania

## Abstract

**Background:**

Effective vaccines deployable globally are needed to control the COVID-19 pandemic. INO-4800, a plasmid DNA vaccine encoding the ancestral SARS-CoV-2 spike (S) protein, has demonstrated safety and immunogenicity. We report the stability of multiple INO-4800 lots used in Phase 3 clinical trials.

**Methods:**

Lots of INO-4800 stored at the test temperatures (storage temperature [2–8°C] and accelerated temperature [25 ± 2°C]) were sampled every 3 months. Sample stability at each timepoint was assessed in terms of 2 parameters: i) purity as measured by DNA isoform homogeneity by capillary gel electrophoresis (CGE) to quantify relative proportions of different plasmid topologies (supercoiled, open circular, and linear), and ii) potency as measured by a cell-based assay for flow cytometric detection of fluorescently labeled S protein.

**Results:**

At 2–8°C, **all** tested lots of INO-4800 retained structural homogeneity for ≥12 months **(Fig. 1A)**. Proportions of circular and supercoiled plasmid isoforms were ≥96% and ≥89%, respectively, remaining above minimum regulatory authority-approved specifications (≥85% for circular and ≥80% for supercoiled isoforms). At 25 ± 2°C, **all** tested lots retained structural homogeneity within specifications for ≥6 months **(Fig. 1B)**, with proportions of circular and supercoiled plasmid isoforms being ≥96% and ≥81%, respectively. Moreover, structural homogeneity of 1/2 (50%) lots was within specification at 9 months at 25 ± 2°C. Potency of **all** tested lots was ≥84% for ≥6 months at both 2–8°C and 25 ± 2°C, remaining above the regulatory authority-approved minimum of 77% **(Figs. 1C and 1D)**. Moreover, 3/3 (100%) of tested lots were within potency specification at 12 months at 2–8°C and 2 lots and 1 lot, respectively, were within specification at 9 and 12 months at 25 ± 2°C. Data from additional timepoints are accruing and will be presented.

**Figure 1:**
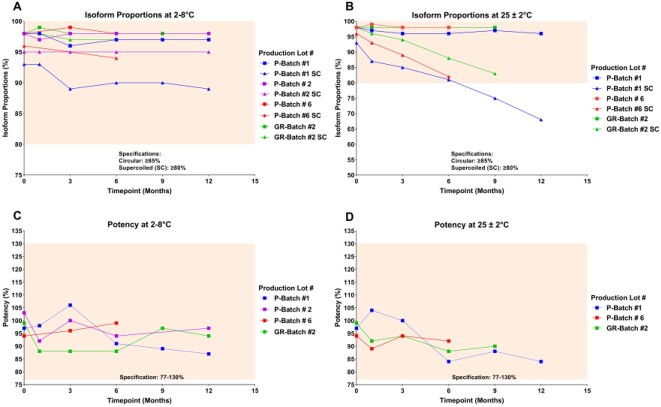
Stability of INO-4800 lots used in Phase 3 trials as measured by isoform homogeneity (A and B) and potency (C and D). Shaded areas represent regulatory authority-approved specifications.

**Conclusion:**

INO-4800 is stable across temperatures ranging from 2°C to 25 ± 2°C and does not require ultra-cold storage. This demonstrated stability confers upon INO-4800 the potential to be deployed globally even in resource-constrained settings lacking cold chain infrastructure, thereby contributing to pandemic preparedness and control.

**Disclosures:**

**Robert J. Juba, Jr., MS Chemical Engineering Practice, BS Chemical Engineering**, Inovio Pharmaceuticals: Employee|Inovio Pharmaceuticals: Stocks/Bonds **Katherine Seals, n/a**, Inovio Pharmaceuticals: Employee|Inovio Pharmaceuticals: Stocks/Bonds **Peggy Kinney, MS Quality Management**, Inovio Pharmaceuticals: Employee|Inovio Pharmaceuticals: Stocks/Bonds.

